# Acute exacerbation of idiopathic pulmonary fibrosis model in the rats using bleomycin and lipopolysaccharides

**DOI:** 10.5455/javar.2023.j669

**Published:** 2023-06-30

**Authors:** Sandy Vitria Kurniawan, Melva Louisa, Jamal Zaini, Silvia Surini, Vivian Soetikno, Puspita Eka Wuyung, Rosemary Ceria Tatap Uli

**Affiliations:** 1 Doctoral Program in Biomedical Sciences, Faculty of Medicine, Universitas Indonesia, Jakarta, Indonesia; 2 Department of Pharmacology and Pharmacy, School of Medicine and Health Sciences, Atma Jaya Catholic University of Indonesia, Jakarta, Indonesia; 3 Department of Pharmacology and Therapeutics, Faculty of Medicine, Universitas Indonesia, Jakarta, Indonesia; 4 Department of Pulmonology and Respiratory Medicine Faculty of Medicine Universitas Indonesia, Persahabatan National Respiratory Referral Hospital, Jakarta, Indonesia.; 5 Laboratory of Pharmaceutics and Pharmaceutical Technology, Faculty of Pharmacy, Universitas Indonesia, Depok, Indonesia; 6 Department of Anatomical Pathology, Faculty of Medicine Universitas Indonesia, Depok, Indonesia; 7 Animal Research Facilities, Indonesian Medical Education and Research Institute, Faculty of Medicine Universitas Indonesia, Depok, Indonesia

**Keywords:** Acute exacerbation, bleomycin, bronchoalveolar lavage fluid, inflammation, idiopathic pulmonary fibrosis, lipopolysaccharides

## Abstract

**Objective:**

This study was conducted to establish a rat model of acute exacerbation of idiopathic pulmonary fibrosis (AE-IPF) using the combination of bleomycin (BLM) and lipopolysaccharides (LPS).

**Materials and Method:**

Twenty-four male Sprague Dawley rats were allocated into two equal groups: the sham or the bleomycin and lipopolysaccharides-induced AE-IPF group (BLM-LPS). On Day 7, BLM intratracheally and LPS intraperitoneally were both used to administer AE-IPF. The BLM-LPS group and its respective sham group were terminated on Days 8, 14, or 21. Samples of bronchoalveolar lavage fluid (BALF) and lungs were taken and investigated for cell count and histopathology.

**Results:**

On Day 8, histological analysis revealed inflammatory cell infiltration with edema and hyaline membrane, and the BALF differential cell count revealed high neutrophil counts. By having a higher collagen density area and Ashcroft modified score than the sham group on Day 14, the BLM-LPS group displayed significantly lower oxygen saturation, alveolar air area, and a fibrotic appearance. However, there was a spontaneous resolution in inflammation and fibrotic appearance on Day 21 after the BLM administration.

**Conclusions:**

By combining BLM and LPS, it was possible to create a successful rat model of AE-IPF. The present model showed the peak exacerbation on Day 8 and the fibrotic peak on Day 14, which gradually improved. The optimal time for the new AE-IPF therapeutic intervention was determined to be between Days 8 and 14.

## Introduction

Idiopathic pulmonary fibrosis (IPF) is a chronic and progressive disease of unknown etiology of the respiratory system that is limited to lung tissue [1]. Patients with IPF may experience a sudden life-threatening event known as an acute exacerbation of the IPF. Acute exacerbations of idiopathic pulmonary fibrosis (AE-IPF) occur 4%–20% of the time, with a life expectancy of 3–4 months [2–4]. According to Japanese IPF treatment guidelines, AE-IPF therapy consists of symptomatic therapy, long-term oxygen therapy, and corticosteroid administration [5,6]. Anti-fibrotic agents such as nintedanib or pirfenidone were used in addition to corticosteroids to improve AE-IPF [7,8]. Long-term use of systemic corticosteroids and anti-fibrosis medications can have serious side effects and has not yielded the best results [9]. Anti-fibrosis medications are costly, and studies found that pirfenidone reduced IPF mortality but had no effect on acute exacerbations [7,10]. As a result, numerous studies have been conducted to identify new drug candidates capable of overcoming this problem.

A suitable animal model is needed to identify a potential new therapy for AE-IPF. An animal model is expected to be able to mimic a condition or disease in humans and its manifestations. Although animal models cannot reproduce all of the variables seen in human illness, they may give insight into the pathobiology of tissue damage, cellular inflammation, immunological control, and the recovery process [11,12]. The American Thoracic Society has recommended the use of mice as IPF animal models rather than rats. Rats, on the other hand, can be used if the use of mice is not feasible due to practical considerations, most notably the limited tissue and blood samples generated from mice. However, a direct comparison of rats and mice demonstrates a similar response to lung injury [11].

Among the many agents to induce IPF, bleomycin-induced lung fibrosis is the most widely used in animal models. Bleomycin (BLM) is an anticancer agent with significant pulmonary toxicity; thus, BLM has been commonly used in preclinical testing [13,14]. Bleomycin-induced pulmonary toxicity cannot be considered an acute exacerbation of lung disease. To induce acute lung injury, lipopolysaccharides (LPS), an endotoxin from gram-negative bacteria, can be used. LPS can be injected directly into the airway or administered systemically via intravenous or intraperitoneal routes. LPS administration causes an inflammatory response, endothelial apoptosis, and cell damage [15,16]. While AE-IPF is a severe condition that may arise in IPF patients, the death rate of individuals suffering from AE-IPF is relatively high, and there is currently no adequate medicine capable of overcoming this problem. The development of AE-IPF medication necessitates the implementation of an animal model of AE-IPF that is simple to produce and can mirror real-world human instances [17]. To date, there have only been a few rat models of AE-IPF. The available animal models have several limitations, including the heterogeneous regions of inflammation in the lung and its reversibility [18–20]. Thus, the present study aimed to determine whether bleomycin-lipopolysaccharide administration in rats could produce a suitable model for AE-IPF.

## Materials and Methods

### Ethical approval

After obtaining ethical clearance from the Ethics Committee of the Faculty of Medicine, Universitas Indonesia (KET-517UN2.F1/ETIK/PPM.00.02/2021), this experiment was performed at the Indonesian Medical Education and Research Institute.

### Chemicals

Bleomycin hydrochloride for injection (Bleocin^®^) was obtained from PT Kalbe Farma, Tbk (Indonesia). LPS from *Escherichia coli* O55:B5 were purchased from Sigma Aldrich, St. Louis, Missouri (cat no. L2880). Ketamine HCl (KTM-100^®^) was purchased from Guardian Pharmatama (Indonesia). Xylazine for injection (Xyla^®^) was purchased from Interchemie (Holland). NaCl (0.9% solution) was purchased from B Braun Pharmaceutical (Indonesia). Natural Buffered Formalin 10% was purchased from BBC Chemical (USA). All other chemicals and solvents were analytical grade.

### Animals and treatments

Male Sprague-Dawley rats (250–300 gm) were obtained from the National Agency for Drug and Food Control’s Animal Breeding Facility at the age of 8–10 weeks. The rats used in the study were acclimatized for 2 weeks before the experiment started. The rats were placed in a room at a temperature of 25°C–27°C and a humidity of 45%–65%, 12-h light and dark cycles, standard pellet food, and unlimited water.

Twenty-four rats were randomly divided into two groups of 12: the sham group and the bleomycin-lipopolysaccharides (BLM-LPS) group. Rats were anesthetized intramuscularly with ketamine (75 mg/kg BW) and xylazine (5 mg/kg BW) before receiving intratracheal administration of saline (sham group) or BLM (BLM-LPS group). The rats in the sham group were incised in the trachea region and stitched back, followed by the administration of 0.9% saline solution intraperitoneally on Day 7. In the BLM-LPS group, 5 mg/kg BW BLM was injected intratracheally with a 30-gauge needle, followed by air. The rats were shaken several times to ensure that BLM was distributed evenly throughout both lung tissues. On Day 7, after BLM, the rats in the BLM-LPS group received an LPS injection (7.5 mg/kg BW) intraperitoneally. Both groups of rats were randomly terminated three times, on Days 8, 14, and 21, to observe differences in disease progression over time following BLM-LPS induction.

On the day of animal sacrifice (Days 8, 14, or 21), rats were anesthetized using ketamine (75 mg/kg BW) and xylazine (5 mg/kg BW) via the intramuscular route. After being anesthetized, oxygen saturation was checked in the rats using an animal pulse oximeter. A probe to measure oxygen saturation was placed on the rats’ leg. Blood samples were collected via heart puncture. The neck and thoracic cavities were opened, a 22-gauge intravenous catheter was injected into the trachea, and the bronchoalveolar lavage fluids were collected. The needle was pulled out, and the plastic tube was left to connect to a syringe containing 2–3 ml of saline. Saline was slowly injected into both lungs. While saline was injected, the rat’s body was slightly lifted, and the rat’s nostrils were closed with a finger to ensure no fluid came out of the nose. This procedure was repeated three times, and the BALF was collected in the same tube. Afterward, the lungs were harvested, rinsed in saline, dried on paper towels, and weighed to calculate the lung/body weight (BW) index. Lungs were immersed in 10% neutral-buffered formalin for histopathological examination.

### BALF examination

Cold centrifugation at 1,000x*g* for 20 min was used to separate the BALF from each rat. Phosphate-buffered saline was used to resuspend sedimentary cell pellets. The procedure was divided into two parts: the cell count procedure and the cytospin procedure. The improved Neubauer counting chamber was used to determine cell counts, and trypan blue exclusion was used to determine viability. For 5 min, the cytospin procedure was performed at 1,500 rpm. Giemsa staining was performed after the cytospin procedure (Cytospin 4 Cytocentrifuge Epredia). The differential cell count was assessed using a light microscope (Leica DM 1000 LED) on Giemsa-stained slides.

### Histopathological examination of Hematoxylin-Eosin (HE) and Masson’s Trichrome-stained lung specimen

The lung tissues were embedded in paraffin wax, sectioned (5 μm), and stained with HE and Masson’s trichrome after being placed in a vial containing 10% neutral-buffered formalin. Tissues were examined using a Leica DM 1000 LED microscope. HE stains were used to examine cell morphology and inflammation. Collagen fibers in tissues were detected using Masson’s trichrome staining. Hubner et al. [21] adapted the Ashcroft score to classify semi-quantitative morphological changes in lung sections. The degree of lung tissue fibrosis was graded on a scale of 0 to 8 using a 200× magnification field (5 pictures for each slide). Pathologists with extensive experience did the scoring. The dominant degree of fibrosis was distinguished as taking up more than half of the field area. The overall score was calculated by averaging the individual scores observed in all microscopic fields.

### Quantification of collagen content

ImageJ 1.53t analysis software was used to determine the degree of fibrosis. Regions of interest (ROIs) were chosen randomly using a 100× magnification and three images per slide. An image analysis application was used to identify regions of green collagen in each ROI using similar contrast, intensity, and color threshold settings [22]. The collagen content was calculated as a percentage of the collagen deposition area (μm^2^), corresponding to the total lung region (μm^2^) in the ROI. The ROI area did not include bronchi and blood vessels [23].

### Quantification of alveolar air area

Alveolar air area was measured as an indirect fibrosis parameter. A white threshold was used to detect the alveolar air area within the ROIs using ImageJ 1.53t analysis software, and the same ROIs were used for counting collagen content. The percentage area (μm^2^) occupied by air was expressed as a percentage of the total lung area (μm^2^) in ROI. The ROI area did not include bronchi and blood vessels [23].

### Data analysis

The mean and standard error of the mean (SEM) were used for presenting the data. Multiple *t*-tests were applied for statistical analysis (GraphPad Prism version 9.5.0). A *p*-value of less than 0.05 was considered significant.

## Results

### Decrease in the BW and arterial oxygen saturation in the rats administered with BLM-LPS

**Figure 1.**
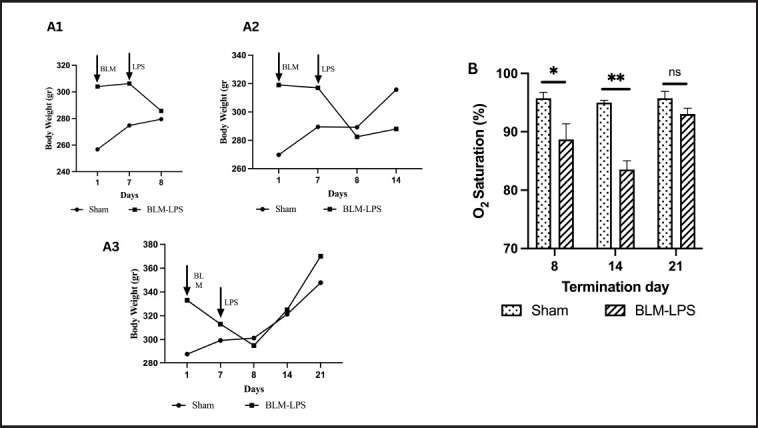
(A) BW of rats in the sham and BLM-LPS-induced groups sacrificed on Days 8 (A1), 14 (A2), and 21 (A3); (B) Oxygen saturation of rats in the sham and BLM-LPS groups sacrificed on Days 8, 14, and 21. BLM: bleomycin; LPS: lipopolysaccharides; *: *p *< 0.05 *vs.* sham group; **: *p *< 0.001 *vs.* sham group; ns = not significant.

Following bleomycin administration, rats given BLM-LPS had a slight decrease in BW compared to rats in the sham group (Fig. 1a). On Day 7, a significant decrease in BW was observed 1 day after LPS injection, which was reversible on Days 14 and 21. (Fig. 1a). On Days 8 and 14, there was a decrease in arterial oxygen saturation along with a decrease in BW, which was improved on Day 21 (Fig. 1b).

### Increase in the lung weight and lung index in the BLM-LPS group

**Figure 2.**
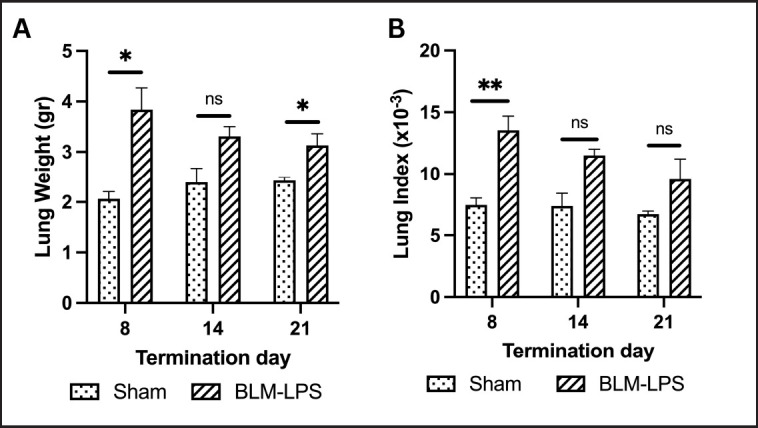
Lung weight and lung index of rats in the sham and BLM-LPS-induced groups sacrificed on Days 8, 14, and 21. BLM: bleomycin; LPS: lipopolysaccharides; *: *p *< 0.05 *vs.* sham group; **: *p *< 0.001 *vs*. sham group; ns = not significant.

The lung index was determined by dividing the lung weight by the total BW of the rats. Higher lung indexes were seen in the BLM-LPS-induced animals compared to the sham group. On Day 8, the rats had the most significant lung index, followed by Days 14 and 21 (Fig. 2).

*Analysis of BALF with neutrophil marked increase in the BLM-LPS group*

**Figure 3.**
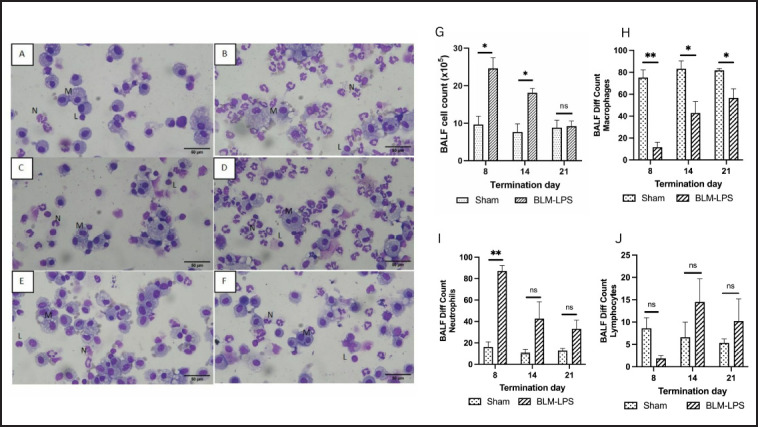
Giemsa staining of BALF cytospin (A–F): rats in the sham group sacrificed on Day 8 (A), rats in the BLM-LPS group sacrificed on Day 8 (B), rats in the sham group sacrificed on Day 14 (C), rats in the BLM-LPS group sacrificed on Day 14 (D), rats in sham group sacrificed on Day 21 (E) rats in the BLM-LPS group sacrificed on Day 21 (F). BALF cell count of rats in the sham and bleomycin-LPS-induced groups sacrificed on Days 8, 14, and 21 (G-J): total cell counts (G); macrophages (H); neutrophils (I) and lymphocytes (J). BLM: bleomycin; LPS: lipopolysaccharides; M: macrophages; N: neutrophils; L; lymphocytes; *: *p *< 0.05 *vs.* sham group; **: *p *< 0.001 *vs.* sham group; ns = not significant.

Cell counts in BALF were examined on the same day as the rat necropsy. The number of cells increased significantly in the BLM-LPS group terminated on Day 8, then decreased slightly in the BLM-LPS group terminated on Day 14, and returned to normal on Day 21 (Fig. 3g).

Most cells in the differential count in the sham group were macrophages, according to BALF examination using cytospin followed by Giemsa staining (Fig. 3A–F, H–J). However, on Day 8 (24 h after LPS administration), in rats induced with BLM and LPS, there was a very high increase in the number of neutrophils. Then, on Day 14, the number of macrophages and neutrophils became more balanced, and on Day 21, the number of neutrophils decreased and the number of macrophages improved.

*Marked inflammatory cell infiltration on Day 8 in the rats administered with BLM-LPS*

**Figure 4.**
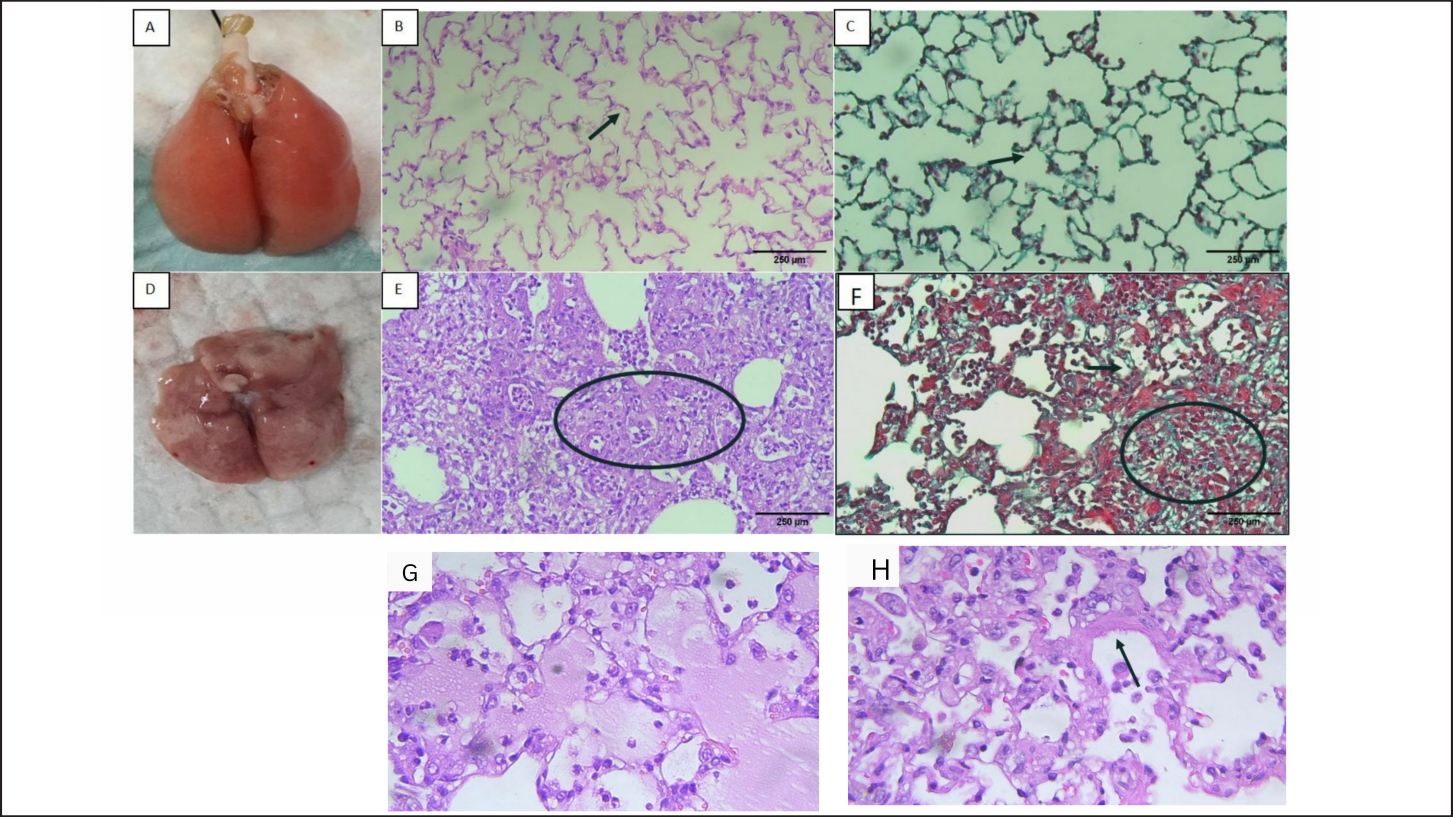
Lungs of the rats in sham (A-C) and BLM-LPS groups (E–H) sacrificed on Day 8: (A) macroscopic appearance of the rat lung in sham group; (B) HE staining of the rat lung in sham group; (C) Masson’s Trichrome staining of the rat lung in the sham group; (D) macroscopic appearance of the rat lung in BLM-LPS group; (E) HE staining of the rat lung in the sham group. The circle shows the alveolus filled with inflammatory cells; (F) Masson’s Trichrome staining of the rat lung in the BLM-LPS group showed lung enlargement. Arrows indicate alveolar septal thickening; circles indicate alveoli that have been covered with green fibrotic tissue; (G) Pulmonary edema fluid in BLM-LPS groups and (G) hyaline membrane formation (arrow). Magnification (A–F): 200×; G-H: 400 ×.

Figure 4 depicts the lungs of rats terminated on Day 8, with macroscopic images of sham rat lungs showing normal lungs (Fig. 4a). The microscopic findings after HE staining showed normal alveolar space and alveolar septal thickness (Fig. 4b), and Masson’s Trichrome image showed a small number of collagen fibers (in green) in the alveolar septum (Fig. 4c). A macroscopic lung image of the rats in the BLM-LPS group is shown in Figure 4D, which shows a hemorrhagic collapsed lung with a rough surface and the development of white or grey fibrous nodules. The HE staining revealed significant inflammatory cell infiltration, thickening of the alveolar septum, and pulmonary edema, and the alveoli were covered by inflammatory cells and a hyaline membrane (Fig. 4e–h). Masson’s trichrome staining reveals a large amount of collagen and confluent fibrotic mass covering 50% of the microscopic field of view and damage to lung structures.

*Large lung fibrotic area on Day 14 in the rats administered with BLM-LPS*

**Figure 5.**
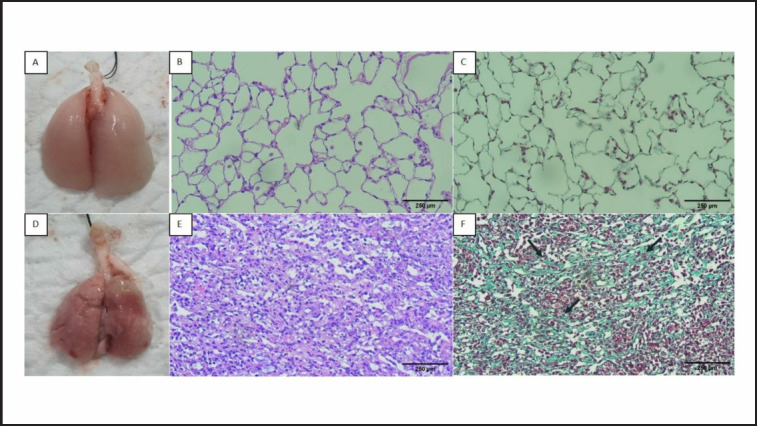
The lung of the rats in the sham group (A–C) and BLM-LPS groups (D–F) terminated on Day 14: (A) macroscopic appearance of the rat lung in the sham group; (B) HE staining of the rat lung in sham group; (C) Masson’s Trichrome staining of the rat lung in the sham group; (D) macroscopic appearance of the rat lung in BLM-LPS group; (E) HE staining of the rat lung in the sham group. (F) Masson’s Trichrome staining of the rat lung in the BLM-LPS group. Arrows indicate fibrotic tissue. Magnification: 200×. BLM: bleomycin; LPS: lipopolysaccharides.

Figure 5 displays the lungs of rats that were terminated on Day 14. The macroscopic view (Fig. 5a) of the lungs of sham rats revealed a normal lung appearance. HE staining (Fig. 5b) revealed normal alveoli with intact alveolar shape, alveolar septal thickness, and collagen fibers in the alveolar septum within normal limits. Masson’s trichrome staining (Fig. 5c) revealed collagen fibers in the alveolar septum within normal limits. Macroscopically, more extensive lung damage was seen in BLM-LPS rats terminated on Day 14 compared to rats terminated on Day 8 with more nodules, hemorrhagic lesions, and rough pulmonary surface areas (Fig. 5d). The inflammatory cell infiltration was widespread on the microscopic image with HE staining (Fig. 5e), covering the alveoli so that the image of the alveoli was invisible. Meanwhile, Masson’s trichrome staining revealed a sizeable fibrotic mass (>50% of the microscopic field of view) (Fig. 5f). The lung structure can no longer be maintained.

*Improvement of inflammation and fibrosis on Day 21 in the rats administered with BLM-LPS*

**Figure 6.**
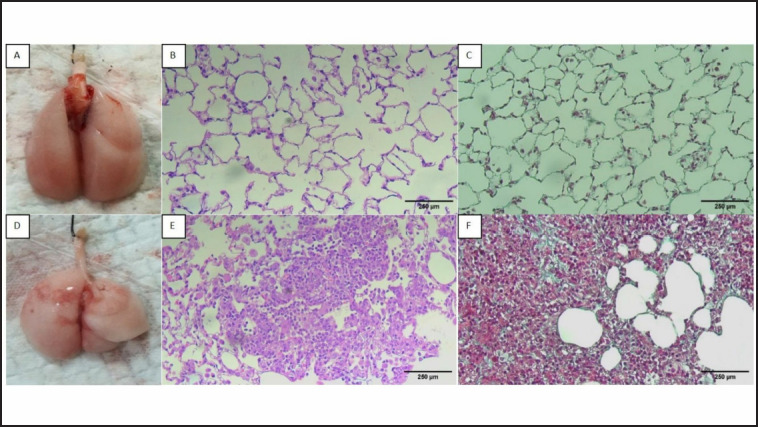
The lung of the rats in the sham group (A-C) and BLM-LPS groups (D-F) terminated on Day 21: (A) macroscopic appearance of the rat lung in the sham group; (B) HE staining of the rat lung in sham group; (C) Masson’s Trichrome staining of the rat lung in the sham group; (D) macroscopic appearance of the rat lung in BLM-LPS group; (E) HE staining of the rat lung in the sham group. (F) Masson’s Trichrome staining of the rat lung in the BLM-LPS group. Magnification: 200×, BLM: bleomycin; LPS: lipopolysaccharides.

Figure 6 illustrates the lungs of rats terminated on Day 21. The sham group rats had a normal lung macroscopic appearance, regular alveolar cavities and alveolar septum, and few collagen fibers within normal limits (Fig. 6a–c). The BLM-LPS group showed macroscopic evidence of lung damage. There were fewer nodules and a smoother surface (Fig. 6d) compared to the findings on Day 14. In addition, we found less inflammatory cell infiltration on HE staining (Figure 6e) compared to the BLM-LPS group terminated on Day 14; however, there was still damage to the alveoli and thickening of the alveolar septum. A confluent fibrotic mass was seen on Masson’s trichrome staining (Fig. 6f) but not in all visual fields (50%), and the lung structures appeared damaged.

*Persistent increase in Ashcroft modified scores and collagen density area as well as a decrease in alveolar air area in the BLM-LPS group on Days 8, 14, and 21*

**Figure 7.**
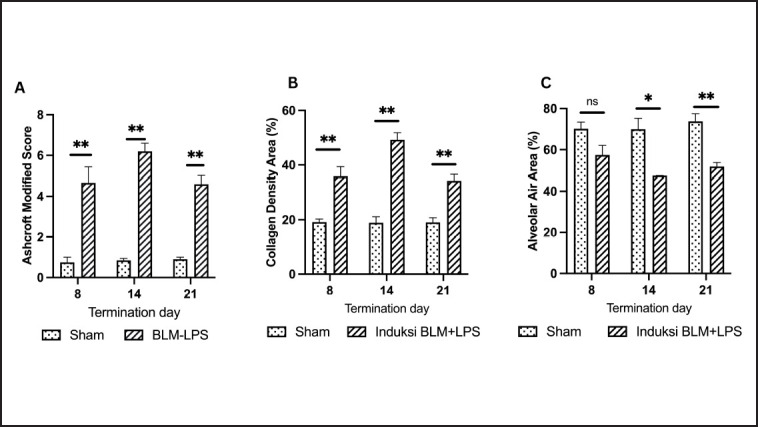
(A) Ashcroft modified Score of rats terminated on Days 8, 14, and 21; (B) collagen density area of rats terminated on Days 8, 14, and 21; (C) alveolar Air Area of rats terminated on Days 8, 14, and 21. BLM: bleomycin; LPS. * : *p *< 0.05 *vs.* sham group; **: *p *< 0.001 *vs.* sham group; ns = not significant.

The Ashcroft modified score increased significantly in all bleomycin-lipopolysaccharide groups on Days 8, 14, and 21 (Fig. 7a). The highest score was found on Day 14, indicating that the fibrotic process had peaked. Although the Ashcroft modified score tended to decrease on Day 21, which reflects fibrosis resolution, the score remained relatively high.

The ImageJ application measured the green-colored areas on lung slides stained with Masson’s trichrome to determine collagen density. The area of collagen density increased with BLM and LPS induction, reaching a peak in rats on Day 14. On Day 21, there was a decrease in collagen density, indicating that the rat lung injury had started to resolve (Fig. 7b).

The alveolar air area can be seen on lung slides stained with Masson’s trichrome in the ImageJ application by measuring the white area. In BLM and LPS-induced rats, alveolar air area decreased for 8, 14, and 21 days, with a maximum decrease at 14 days (Fig. 7c).

## Discussion

Patients with IPF may experience life-threatening acute exacerbations called AE-IPF. AE-IPF has a poor prognosis and a high morbidity and mortality rate, which can lead to increased hospitalization rates. Reduced lung function, impaired oxygenation, and a high fibrosis score increase the risk of death in patients with AE-IPF [24].

The molecular pathogenesis of IPF is not fully understood. However, epithelial injury and poor wound healing are thought to play essential roles in this disease, in addition to chronic inflammation. Old age, exposure to cigarettes and asbestos, comorbid diseases, such as diabetes mellitus, gastroesophageal reflux, and obstructive sleep apnea, and infections are all risk factors for IPF [25].

AE-IPF pathobiology is a combination of chronic elements such as epithelial cell malfunction, fibroblast buildup and activation, and other significant factors such as acute stress and acute lung damage. Critical events such as infection or microaspiration induce acute broad lung damage, characterized by the production of hyaline membranes and interstitial edema in the early stages [1].

The lack of animal models that mimic AE-IPF cases creates barriers to advancing AE-IPF research. Rats have been used in drug research for a long time because they have larger bodies and more blood volume than mice. A suitable rat model enables a more thorough examination of test parameters. As a result, we intend to create an AE-IPF rat animal model for future research.

The use of intratracheal BLM to create pulmonary fibrosis animal models has been established and widely used in various studies [12,26]. BLM is a cell-cycle chemotherapeutic substance that acts in the G2 and M phases. BLM forms free radicals after binding to iron, causing DNA chain breaks and cell death. BLM rapidly deactivates in the liver and kidneys due to the enzyme BLM hydrolase hydrolyzing and eliminating it through the kidneys. BLM targets the skin and lungs due to the low level of BLM hydrolase. BLM toxicity in the lungs can cause fibrosis and lung injury. However, pulmonary toxicity caused by BLM is not an acute manifestation of lung disease. Thereby, LPS can induce acute lung injury [26,27].

In this study, the administration of LPS on Day 7 was intended to mimic AE-IPF in humans by acting as an infection that causes an acute exacerbation state. Our study showed that using BLM followed by LPS resulted in a good model of AE-IPF with an optimal window between Days 8 and 14 after BLM administration. The administration of BLM alone had little effect on the BW of the rats. However, drastic weight loss was observed in the rats after administration of LPS, which was intended to mimic acute exacerbations; however, the effect was transient and tended to resolve at Days 14 and 21. LPS (LPS, an outer membrane component of Gram-negative bacteria) is the primary trigger of lung inflammation in experimental animals. It is commonly accepted as a reliable inducer of acute exacerbation in lung diseases [28,29].

The research findings were consistent with those of Miyamoto et al. [18] who reported that the group given BLM-LPS induction lost significantly more weight than those given only BLM or LPS [18]. Another study by Akhter et al. found that BW decreased within 24 h of administering LPS to induce lung inflammation in mice [30]. In addition, Kim et al. [31] demonstrated that intraperitoneal injection of LPS increased herpes virus entry mediator (HVEM) in the hypothalamus, causing anorexia and weight loss. HVEM-activated transcription factors such as nuclear factor kappa B and activator protein-1 regulate gene expression in infections’ inflammatory and acute phases. All these inflammatory responses result in anorexia-like behavior. The rapidly occurring migration of inflammatory cells into lung tissue after intraperitoneal LPS injection, followed by subsequent fibrosis, explains the systemic consequences. LPS triggers massive inflammatory mediators to be released into the systemic circulation, which leads to indirect lung injury and other systemic effects [28].

The oxygen saturation was the lowest in BLM-LPS-induced rats on Day 14, yet it improved on Day 21. The improvement in oxygen saturations indicates that spontaneous resolution has occurred as time has elapsed. This is consistent with the findings of Miyamoto et al. [18], who discovered that 14 days after LPS administration, pO_2_ and pCO_2_ had returned to normal, indicating that breathing had improved. Macroscopic and microscopic examinations of BLM-LPS rats terminated on Day 14 revealed more extensive lung structural damage, with inflammatory cell infiltration and fibrotic tissue overlying the alveolus. However, when BLM-LPS rats were terminated on Day 21, the inflammatory cell infiltration in the lungs decreased, as did the fibrotic tissue. This image shows that there has been an improvement on Day 21, while the fibrotic peak occurs on Day 14.

Despite some discrepancies in the results, numerous studies have shown that the acute IPF worsening brought on by BLM-LPS is partially reversible [28, 32, 33]. The distinct modulation of BLM and LPS-related signaling pathways throughout the injury period may explain the reversibility of the induction. Animals have an important feature in the fibrosis repair process, which includes extracellular matrix component deposition. Delayed TGF-b and collagen deposition over time might slow the inflammation process in fibrogenesis [32, 34]. Evidence has demonstrated that LPS exposure alters signaling pathways over time. TLR-4, which functions as an LPS receptor, is consistently elevated for many days after LPS injections. TLR-4 recognizes LPS via CD14 and MD-2, which suggests a unique sequence of activation [35]. CD14 gene expression in the lung quickly increases to peak levels 3 h after intranasal LPS challenge, then decreases to baseline levels 4 days later [15, 18, 28].

This study found an increase in lung index in BLM-LPS rats terminated on Days 8, 14, and 21, which was due to the increment in the weight of the rat lungs. This suggests that inflammatory cell infiltration and fibrosis in the lung increase lung weight. In an animal model of acute lung injury, Song et al. [36] reported an increase in lung weight and index. Cowley et al. [37] stated that BLM-induced mice increased lung weight due to increased inflammation, fluid accumulation, and lung fibrosis. The lung weight ratio per BW index will rise as lung weight increases. According to Wahlstrom et al. [38], lung weight increases in cases of acute lung injury, and lung weight can indicate a substance’s toxic effect.

The findings confirmed that when LPS is administered on Day 7, it causes an acute exacerbation condition with a peak effect on Day 8. The BALF cell count dramatically increases on Day 14 and gradually decreases on Day 21. On Day 8, the differential BALF count with Giemsa stain revealed that neutrophils were elevated following LPS administration. This indicates a severe infection. This is similar to the AE-IPF condition in humans, where neutrophils are reported to be the predominant cell type in BALF. According to Schupp et al. [39], there was an increase in neutrophil percentage in IPF patients during A.E. compared to more stable patients and a decrease in lymphocyte and macrophage percentage. Kimura et al. [19] also noted a significant increase in neutrophils after LPS administration in the BLM-LPS-induced group. On Day 14, the number of neutrophils decreased and reached the same level as the number of macrophages. On Day 21, macrophages continued to increase.

In HE staining of BLM-LPS rats on the eighth day, there was an infiltration of inflammatory cells covering the alveoli, pulmonary edema, and the formation of hyaline membranes, which corresponded to the description of AE-IPF cases in humans [1]. Administration of LPS causes an acute lung injury event characterized by inflammation, which is mediated by an increase in neutrophils. This damages the alveolar-capillary barrier, increasing capillary permeability and causing intra-alveolar bleeding and edema. Gas exchange in the alveoli is disrupted, as is the formation of hyaline membranes on the alveolar walls [19,40].

The limitation of this study includes the relatively short period of time over which we monitored the resolution of pulmonary fibrosis. We suggest that the study be extended to 28 days after BLM injection. Furthermore, further tests may be performed in animal models to measure the amount of inflammation and fibrosis. Though BLM-LPS-induced rats are a good model for understanding the progressivity of the disease, they could not fully replicate the irreversible course of fibrosis seen in patients [32]. Therefore, while using this model for medication intervention research, it is important to keep in mind that one of the most important characteristics of human IPF is lacking in animals.

Based on all the above findings, acute exacerbations occurred on Day 8, 1 day after LPS administration, with fibrosis peaking on Day 14. These findings were supported by an increase in collagen density area and a decrease in alveolar air area found in BLM-LPS rats terminated on Day 14. The disadvantage of using BLM to induce IPF is the possibility of spontaneous resolution [33]. According to Izbicki et al. [32], the fibrotic peak was discovered on Day 14 after BLM administration, and the outcome on Day 21 was highly variable, with resolution possible. However, a researcher claimed that the new resolution might take effect on Day 28 [11].

## Conclusion

The rat animal model of AE-IPF was successfully obtained by administering a combination of BLM and LPS. In the present rat AE-IPF model, a new intervention candidate can be administered between Days 8 and 14 when there is an acute exacerbation and fibrotic peak, and no spontaneous resolution has occurred between the periods.

## List of abbreviations

AE-IPF, Acute Exacerbation of Idiopathic Pulmonary Fibrosis; BALF, Broncho Alveolar Lavage Fluid; BLM, Bleomycin; BW, Body Weight; HE, Hematoxylin-Eosin; HVEM, Herpes Virus Entry Mediator; IPF, Idiopathic Pulmonary Fibrosis; LPS, Lipopolysaccharide; ROIs, Regions of interest; SEM, Standard Error of the Mean.
